# 1,8,16,23-Tetra­kis(2-cyano­benz­yl)bis-*p*-xylylbis-*m*-xylyldiamine

**DOI:** 10.1107/S1600536808033473

**Published:** 2008-10-18

**Authors:** Hong-Ye Bai, Hua Wu, Jian-Fang Ma

**Affiliations:** a Department of Chemistry, Northeast Normal University, Changchun 130024, People’s Republic of China

## Abstract

The title compound {systematic name: 2,2′,2′′,2′′′-[3,7,11,15-tetra­aza-1(1,4),5(1,3),9(1,4),13(1,3)-tetra­benzena­cyclo­hexadeca­phane-3,7,11,15-tetra­yltetra­methyl­ene]tetra­benzonitrile}, C_64_H_56_N_8_, is a centrosymmetric macrocycle that is consolidated into the crystal structure by C—H⋯π inter­actions.

## Related literature

For synthesis, see: Chen & Martell (1991 ▶). For related literature, see: Vigato & Tamburini (2004 ▶). For related structures, see: Chen & Martell (1991 ▶); Comba *et al.* (2001 ▶).
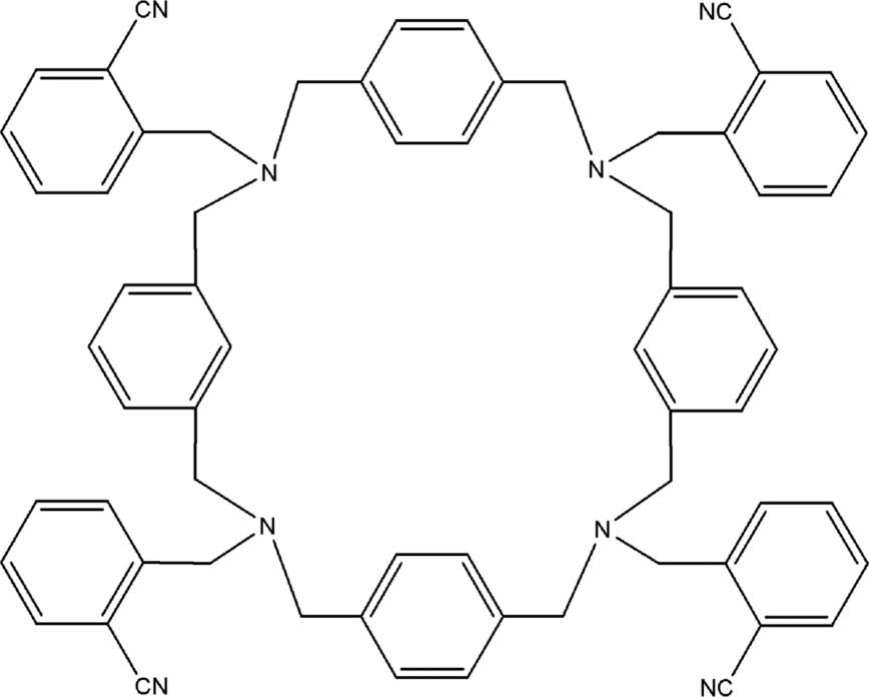


## Experimental

### 

#### Crystal data

C_64_H_56_N_8_
*M* *_r_* = 937.17
Triclinic, 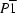

*a* = 9.084 (5) Å
*b* = 10.999 (8) Å
*c* = 14.160 (8) Å
α = 73.26 (2)°
β = 73.012 (19)°
γ = 83.40 (2)°
*V* = 1295.0 (14) Å^3^
*Z* = 1
Mo *K*α radiation
μ = 0.07 mm^−1^
*T* = 293 (2) K
0.21 × 0.19 × 0.17 mm

#### Data collection

Rigaku R-AXIS RAPID diffractometer
Absorption correction: multi-scan (*ABSCOR*; Higashi, 1995 ▶) *T*_min_ = 0.975, *T*_max_ = 0.983
12846 measured reflections
5868 independent reflections
2846 reflections with *I* > 2σ(*I*)
*R*_int_ = 0.045

#### Refinement

*R*[*F* ^2^ > 2σ(*F* ^2^)] = 0.073
*wR*(*F* ^2^) = 0.188
*S* = 1.04
5868 reflections
325 parameters
H-atom parameters constrained
Δρ_max_ = 0.29 e Å^−3^
Δρ_min_ = −0.20 e Å^−3^

### 

Data collection: *PROCESS-AUTO* (Rigaku, 1998 ▶); cell refinement: *PROCESS-AUTO*; data reduction: *PROCESS-AUTO*; program(s) used to solve structure: *SHELXS97* (Sheldrick, 2008 ▶); program(s) used to refine structure: *SHELXL97* (Sheldrick, 2008 ▶); molecular graphics: *SHELXTL-Plus* (Sheldrick, 2008 ▶); software used to prepare material for publication: *SHELXL97*.

## Supplementary Material

Crystal structure: contains datablocks global, I. DOI: 10.1107/S1600536808033473/tk2307sup1.cif

Structure factors: contains datablocks I. DOI: 10.1107/S1600536808033473/tk2307Isup2.hkl

Additional supplementary materials:  crystallographic information; 3D view; checkCIF report

## Figures and Tables

**Table 1:** Hydrogen-bond geometry (Å, °) *Cg*1 and *Cg*2 are the centroids of the C3–C8 and C26–C31 rings, respectively.

*D*—H⋯*A*	*D*—H	H⋯*A*	*D*⋯*A*	*D*—H⋯*A*
C30—H30⋯*Cg*1^i^	0.93	2.60	3.451 (4)	152
C2—H2*A*⋯*Cg*2^ii^	0.97	2.97	3.937 (4)	177

Symmetry codes: (i) 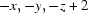; (ii) 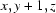.

**Fig. 1.**
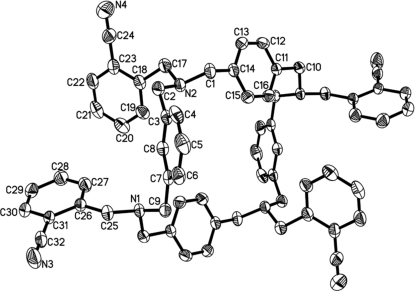
Molecular structure of (I). Displacement ellipsoids are drawn at the 30% probability level and H atoms are omitted for clarity. The unlablled atoms are related by -x+1, -y+1, -z+1.

**Fig. 2.**
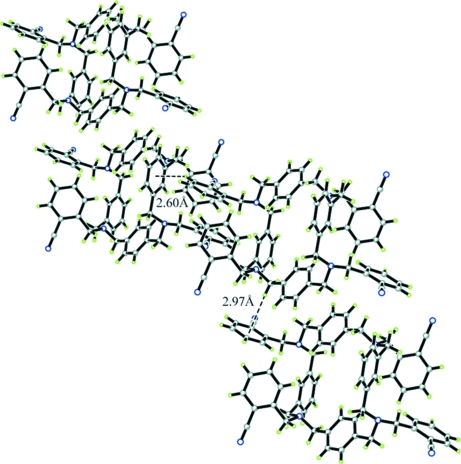
The C—H···π interactions in (I).

**Fig. 3.**
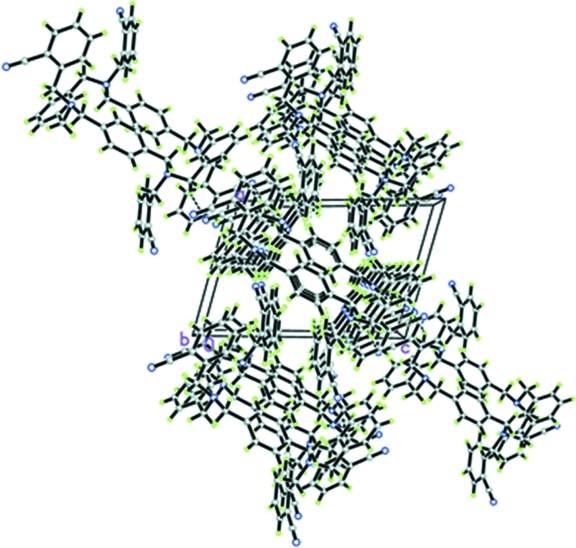
A view down the b-axis of the unit cell contents for (I).

## supplementary crystallographic information

### Comment

Macrocyclic compounds and their derivatives have attracted much attention recently owing to their applications in biochemistry, materials science, catalysis, encapsulation, activation, transport and separation phenomena, hydrometallurgy, *etc*. (Vigato & Tamburini, 2004). As an extension of our research on the macrocyclic derivatives, compound (I) was synthesized and its crystal structure determined, Fig. 1.

Bond lengths and bond angles found for (I) are within normal ranges for related crystal structures (Comba *et al.*, 2001). The crystal structure is stabilized by C—H···π packing interactions, Table 1 and Figs 2 & 3; Cg1 and Cg2 are the centroids of the C3–C8 and C26–C31 rings, respectively.

### Experimental

All chemicals were obtained from commercial sources and used without further purification except for bis-*p*-xylyl-bis-*m*-xylyldiamine which was synthesized according to the literature method (Chen & Martell, 1991). A mixture of bis-*p*-xylyl-bis-*m*-xylyldiamine (0.472 g, 1 mmol) and K_2_CO_3_ (1.00 g) in acetonitrile (30 ml) was stirred for 4 h. 2-Cyanobenzyl chloride (1 mmol) was then added and the solution refluxed for 20 h. After completion of the reaction, the reaction mixture was filtered and the filtrate was evaporated under vacuum. Colorless crystals suitable for X-ray diffraction were obtained by slow evaporation of an acetone solution of (I) after several days at room temperature.

### Refinement

H atoms were treated as riding with C—H = 0.93 - 0.97 Å (CH), and with *U*_iso_ = 1.2*U*_eq_(C).

### Crystal data

**Table:**

C_64_H_56_N_8_	*V* = 1295.0 (14) Å^3^
*M**_r_* = 937.17	*Z* = 1
Triclinic, *P*1	*F*(000) = 496
Hall symbol: -P 1	*D*_x_ = 1.202 Mg m^−^^3^
*a* = 9.084 (5) Å	Mo *K*α radiation, λ = 0.71069 Å
*b* = 10.999 (8) Å	µ = 0.07 mm^−^^1^
*c* = 14.160 (8) Å	*T* = 293 K
α = 73.26 (2)°	Block, colorless
β = 73.012 (19)°	0.21 × 0.19 × 0.17 mm
γ = 83.40 (2)°	

### Data collection

**Table:**

Rigaku R-AXIS RAPID diffractometer	5868 independent reflections
Radiation source: fine-focus sealed tube	2846 reflections with *I* > 2σ(*I*)
graphite	*R*_int_ = 0.045
Detector resolution: 10.0 pixels mm^-1^	θ_max_ = 27.5°, θ_min_ = 3.0°
ω scans	*h* = −10→11
Absorption correction: multi-scan (ABSCOR; Higashi, 1995)	*k* = −14→14
*T*_min_ = 0.975, *T*_max_ = 0.983	*l* = −17→18
12846 measured reflections	

### Refinement

**Table:**

Refinement on *F*^2^	Primary atom site location: structure-invariant direct methods
Least-squares matrix: full	Secondary atom site location: difference Fourier map
*R*[*F*^2^ > 2σ(*F*^2^)] = 0.073	Hydrogen site location: inferred from neighbouring sites
*wR*(*F*^2^) = 0.188	H-atom parameters constrained
*S* = 1.04	*w* = 1/[σ^2^(*F*_o_^2^) + (0.072*P*)^2^ + 0.2406*P*] where *P* = (*F*_o_^2^ + 2*F*_c_^2^)/3
5868 reflections	(Δ/σ)_max_ < 0.001
325 parameters	Δρ_max_ = 0.29 e Å^−^^3^
0 restraints	Δρ_min_ = −0.20 e Å^−^^3^

### Special details

**Table:**

Geometry. All e.s.d.'s (except the e.s.d. in the dihedral angle between two l.s. planes) are estimated using the full covariance matrix. The cell e.s.d.'s are taken into account individually in the estimation of e.s.d.'s in distances, angles and torsion angles; correlations between e.s.d.'s in cell parameters are only used when they are defined by crystal symmetry. An approximate (isotropic) treatment of cell e.s.d.'s is used for estimating e.s.d.'s involving l.s. planes.
Refinement. Refinement of *F*^2^ against ALL reflections. The weighted *R*-factor *wR* and goodness of fit *S* are based on *F*^2^, conventional *R*-factors *R* are based on *F*, with *F* set to zero for negative *F*^2^. The threshold expression of *F*^2^ > σ(*F*^2^) is used only for calculating *R*-factors(gt) *etc*. and is not relevant to the choice of reflections for refinement. *R*-factors based on *F*^2^ are statistically about twice as large as those based on *F*, and *R*- factors based on ALL data will be even larger.

### Fractional atomic coordinates and isotropic or equivalent isotropic displacement parameters (Å^2^)

**Table:**

	*x*	*y*	*z*	*U*_iso_*/*U*_eq_	
C1	0.2541 (3)	0.7033 (3)	0.7026 (2)	0.0587 (7)	
H1A	0.3350	0.6744	0.7368	0.070*	
H1B	0.1856	0.7611	0.7371	0.070*	
C2	0.1184 (3)	0.5227 (3)	0.8221 (2)	0.0580 (7)	
H2A	0.0346	0.4692	0.8316	0.070*	
H2B	0.0791	0.5823	0.8632	0.070*	
C3	0.2436 (3)	0.4416 (2)	0.85999 (19)	0.0514 (7)	
C4	0.2980 (4)	0.4604 (3)	0.9363 (2)	0.0685 (8)	
H4	0.2593	0.5290	0.9633	0.082*	
C5	0.4080 (4)	0.3798 (3)	0.9728 (3)	0.0793 (10)	
H5	0.4430	0.3942	1.0240	0.095*	
C6	0.4666 (3)	0.2779 (3)	0.9339 (2)	0.0648 (8)	
H6	0.5409	0.2236	0.9592	0.078*	
C7	0.4163 (3)	0.2554 (3)	0.85783 (19)	0.0499 (6)	
C8	0.3047 (3)	0.3377 (2)	0.82186 (19)	0.0495 (6)	
H8	0.2697	0.3230	0.7707	0.059*	
C9	0.4840 (3)	0.1459 (3)	0.8133 (2)	0.0548 (7)	
H9A	0.5684	0.1758	0.7528	0.066*	
H9B	0.5259	0.0820	0.8628	0.066*	
C10	0.5508 (3)	0.9901 (2)	0.2845 (2)	0.0554 (7)	
H10A	0.4759	1.0474	0.2558	0.066*	
H10B	0.6264	1.0412	0.2901	0.066*	
C11	0.4700 (3)	0.9112 (2)	0.39063 (19)	0.0497 (6)	
C12	0.3422 (3)	0.9611 (3)	0.4501 (2)	0.0602 (7)	
H12	0.3026	1.0412	0.4231	0.072*	
C13	0.2726 (3)	0.8938 (3)	0.5488 (2)	0.0600 (8)	
H13	0.1867	0.9299	0.5871	0.072*	
C14	0.3260 (3)	0.7751 (3)	0.5925 (2)	0.0502 (6)	
C15	0.4526 (3)	0.7244 (3)	0.5325 (2)	0.0584 (7)	
H15	0.4907	0.6437	0.5592	0.070*	
C16	0.5235 (3)	0.7914 (3)	0.4334 (2)	0.0570 (7)	
H16	0.6089	0.7551	0.3949	0.068*	
C17	0.0341 (3)	0.6353 (3)	0.6741 (2)	0.0650 (8)	
H17A	0.0640	0.6999	0.6095	0.078*	
H17B	−0.0426	0.6735	0.7220	0.078*	
C18	−0.0369 (3)	0.5274 (3)	0.6582 (2)	0.0600 (7)	
C19	0.0540 (4)	0.4340 (3)	0.6196 (2)	0.0643 (8)	
H19	0.1600	0.4334	0.6088	0.077*	
C20	−0.0083 (4)	0.3423 (3)	0.5969 (2)	0.0792 (10)	
H20	0.0554	0.2816	0.5691	0.095*	
C21	−0.1693 (5)	0.3400 (3)	0.6158 (3)	0.0818 (10)	
H21	−0.2118	0.2776	0.6002	0.098*	
C22	−0.2617 (4)	0.4274 (3)	0.6561 (3)	0.0772 (9)	
H22	−0.3679	0.4252	0.6690	0.093*	
C23	−0.1967 (3)	0.5224 (3)	0.6789 (2)	0.0632 (8)	
C24	−0.2924 (4)	0.6135 (3)	0.7200 (3)	0.0710 (9)	
C25	0.2686 (3)	0.0107 (3)	0.8774 (2)	0.0560 (7)	
H25A	0.2388	0.0577	0.9289	0.067*	
H25B	0.3237	−0.0663	0.9042	0.067*	
C26	0.1259 (3)	−0.0250 (2)	0.8603 (2)	0.0504 (6)	
C27	0.0631 (3)	0.0487 (3)	0.7840 (2)	0.0677 (8)	
H27	0.1111	0.1227	0.7409	0.081*	
C28	−0.0698 (3)	0.0151 (3)	0.7699 (3)	0.0784 (10)	
H28	−0.1100	0.0662	0.7178	0.094*	
C29	−0.1422 (3)	−0.0942 (3)	0.8331 (3)	0.0726 (9)	
H29	−0.2307	−0.1176	0.8232	0.087*	
C30	−0.0843 (3)	−0.1679 (3)	0.9101 (2)	0.0638 (8)	
H30	−0.1338	−0.2411	0.9534	0.077*	
C31	0.0486 (3)	−0.1339 (2)	0.9241 (2)	0.0516 (7)	
C32	0.1041 (3)	−0.2134 (3)	1.0065 (3)	0.0674 (8)	
N1	0.3723 (2)	0.08726 (19)	0.78530 (15)	0.0472 (5)	
N2	0.1680 (2)	0.5945 (2)	0.71309 (16)	0.0511 (6)	
N3	0.1478 (4)	−0.2784 (3)	1.0735 (3)	0.0995 (10)	
N4	−0.3737 (4)	0.6894 (4)	0.7527 (3)	0.1080 (11)	

### Atomic displacement parameters (Å^2^)

**Table:**

	*U*^11^	*U*^22^	*U*^33^	*U*^12^	*U*^13^	*U*^23^
C1	0.0600 (16)	0.0602 (18)	0.0538 (16)	−0.0240 (14)	−0.0019 (13)	−0.0173 (14)
C2	0.0576 (17)	0.0523 (17)	0.0537 (16)	−0.0098 (13)	−0.0020 (13)	−0.0086 (14)
C3	0.0557 (16)	0.0462 (16)	0.0452 (15)	−0.0195 (12)	−0.0032 (12)	−0.0054 (12)
C4	0.091 (2)	0.0562 (19)	0.0636 (19)	−0.0270 (17)	−0.0185 (17)	−0.0172 (16)
C5	0.100 (3)	0.079 (2)	0.076 (2)	−0.035 (2)	−0.042 (2)	−0.017 (2)
C6	0.0634 (18)	0.068 (2)	0.0703 (19)	−0.0209 (15)	−0.0335 (15)	−0.0071 (16)
C7	0.0452 (14)	0.0513 (16)	0.0501 (15)	−0.0185 (12)	−0.0111 (12)	−0.0041 (13)
C8	0.0525 (15)	0.0523 (16)	0.0426 (14)	−0.0136 (12)	−0.0107 (12)	−0.0089 (12)
C9	0.0454 (14)	0.0527 (17)	0.0609 (17)	−0.0097 (12)	−0.0132 (13)	−0.0049 (13)
C10	0.0556 (16)	0.0425 (15)	0.0572 (16)	−0.0061 (12)	−0.0049 (13)	−0.0056 (13)
C11	0.0474 (14)	0.0432 (15)	0.0530 (16)	−0.0099 (11)	−0.0060 (12)	−0.0090 (13)
C12	0.0643 (17)	0.0432 (16)	0.0645 (18)	0.0013 (13)	−0.0060 (15)	−0.0142 (14)
C13	0.0529 (16)	0.0539 (18)	0.0624 (18)	−0.0030 (13)	0.0020 (14)	−0.0168 (15)
C14	0.0516 (15)	0.0484 (16)	0.0499 (15)	−0.0155 (12)	−0.0068 (12)	−0.0139 (13)
C15	0.0598 (17)	0.0494 (16)	0.0565 (17)	−0.0009 (13)	−0.0111 (14)	−0.0047 (13)
C16	0.0537 (16)	0.0557 (18)	0.0501 (16)	0.0009 (13)	−0.0028 (13)	−0.0093 (14)
C17	0.0586 (17)	0.0560 (18)	0.081 (2)	−0.0091 (14)	−0.0219 (15)	−0.0129 (16)
C18	0.0638 (18)	0.0516 (17)	0.0633 (18)	−0.0141 (14)	−0.0229 (14)	−0.0029 (14)
C19	0.0703 (19)	0.0523 (18)	0.077 (2)	−0.0072 (15)	−0.0324 (16)	−0.0137 (16)
C20	0.105 (3)	0.060 (2)	0.074 (2)	−0.0093 (19)	−0.033 (2)	−0.0090 (17)
C21	0.105 (3)	0.061 (2)	0.084 (2)	−0.034 (2)	−0.038 (2)	−0.0013 (18)
C22	0.071 (2)	0.064 (2)	0.084 (2)	−0.0225 (17)	−0.0196 (18)	0.0067 (18)
C23	0.0585 (17)	0.0620 (19)	0.0653 (19)	−0.0165 (15)	−0.0245 (15)	0.0020 (15)
C24	0.0563 (19)	0.077 (2)	0.082 (2)	−0.0035 (17)	−0.0257 (17)	−0.0179 (19)
C25	0.0569 (16)	0.0549 (17)	0.0497 (15)	−0.0153 (13)	−0.0077 (13)	−0.0060 (13)
C26	0.0467 (14)	0.0479 (16)	0.0516 (15)	−0.0080 (12)	−0.0044 (12)	−0.0125 (13)
C27	0.0641 (18)	0.0572 (19)	0.073 (2)	−0.0102 (15)	−0.0192 (16)	0.0002 (16)
C28	0.0607 (19)	0.075 (2)	0.094 (3)	−0.0022 (17)	−0.0288 (18)	−0.007 (2)
C29	0.0494 (17)	0.075 (2)	0.093 (2)	−0.0097 (16)	−0.0119 (17)	−0.026 (2)
C30	0.0489 (16)	0.0608 (19)	0.071 (2)	−0.0116 (14)	−0.0001 (15)	−0.0135 (16)
C31	0.0428 (14)	0.0478 (16)	0.0543 (16)	−0.0078 (12)	0.0019 (12)	−0.0116 (13)
C32	0.0583 (18)	0.061 (2)	0.070 (2)	−0.0208 (15)	−0.0060 (16)	−0.0015 (17)
N1	0.0459 (11)	0.0448 (12)	0.0458 (12)	−0.0136 (9)	−0.0057 (9)	−0.0066 (10)
N2	0.0495 (12)	0.0473 (13)	0.0531 (13)	−0.0104 (10)	−0.0115 (10)	−0.0073 (10)
N3	0.092 (2)	0.090 (2)	0.094 (2)	−0.0329 (17)	−0.0286 (18)	0.0232 (19)
N4	0.080 (2)	0.115 (3)	0.132 (3)	0.004 (2)	−0.034 (2)	−0.036 (2)

### Geometric parameters (Å, °)

**Table:**

C1—N2	1.453 (3)	C16—H16	0.9300
C1—C14	1.515 (4)	C17—N2	1.452 (3)
C1—H1A	0.9700	C17—C18	1.514 (4)
C1—H1B	0.9700	C17—H17A	0.9700
C2—N2	1.477 (3)	C17—H17B	0.9700
C2—C3	1.491 (4)	C18—C19	1.378 (4)
C2—H2A	0.9700	C18—C23	1.399 (4)
C2—H2B	0.9700	C19—C20	1.365 (4)
C3—C4	1.386 (4)	C19—H19	0.9300
C3—C8	1.393 (4)	C20—C21	1.411 (5)
C4—C5	1.373 (4)	C20—H20	0.9300
C4—H4	0.9300	C21—C22	1.343 (5)
C5—C6	1.375 (4)	C21—H21	0.9300
C5—H5	0.9300	C22—C23	1.412 (4)
C6—C7	1.378 (4)	C22—H22	0.9300
C6—H6	0.9300	C23—C24	1.392 (5)
C7—C8	1.390 (4)	C24—N4	1.156 (4)
C7—C9	1.506 (4)	C25—N1	1.459 (3)
C8—H8	0.9300	C25—C26	1.499 (4)
C9—N1	1.461 (3)	C25—H25A	0.9700
C9—H9A	0.9700	C25—H25B	0.9700
C9—H9B	0.9700	C26—C27	1.379 (4)
C10—N1^i^	1.460 (3)	C26—C31	1.394 (4)
C10—C11	1.517 (4)	C27—C28	1.385 (4)
C10—H10A	0.9700	C27—H27	0.9300
C10—H10B	0.9700	C28—C29	1.377 (4)
C11—C12	1.380 (3)	C28—H28	0.9300
C11—C16	1.381 (4)	C29—C30	1.360 (4)
C12—C13	1.377 (4)	C29—H29	0.9300
C12—H12	0.9300	C30—C31	1.387 (4)
C13—C14	1.374 (4)	C30—H30	0.9300
C13—H13	0.9300	C31—C32	1.427 (4)
C14—C15	1.382 (4)	C32—N3	1.154 (4)
C15—C16	1.383 (4)	N1—C10^i^	1.460 (3)
C15—H15	0.9300		
			
N2—C1—C14	113.9 (2)	C11—C16—H16	119.4
N2—C1—H1A	108.8	C15—C16—H16	119.4
C14—C1—H1A	108.8	N2—C17—C18	112.8 (2)
N2—C1—H1B	108.8	N2—C17—H17A	109.0
C14—C1—H1B	108.8	C18—C17—H17A	109.0
H1A—C1—H1B	107.7	N2—C17—H17B	109.0
N2—C2—C3	113.7 (2)	C18—C17—H17B	109.0
N2—C2—H2A	108.8	H17A—C17—H17B	107.8
C3—C2—H2A	108.8	C19—C18—C23	118.2 (3)
N2—C2—H2B	108.8	C19—C18—C17	121.1 (3)
C3—C2—H2B	108.8	C23—C18—C17	120.7 (3)
H2A—C2—H2B	107.7	C20—C19—C18	121.5 (3)
C4—C3—C8	117.4 (3)	C20—C19—H19	119.3
C4—C3—C2	123.0 (3)	C18—C19—H19	119.3
C8—C3—C2	119.5 (2)	C19—C20—C21	119.8 (3)
C5—C4—C3	121.2 (3)	C19—C20—H20	120.1
C5—C4—H4	119.4	C21—C20—H20	120.1
C3—C4—H4	119.4	C22—C21—C20	120.3 (3)
C4—C5—C6	120.3 (3)	C22—C21—H21	119.9
C4—C5—H5	119.9	C20—C21—H21	119.9
C6—C5—H5	119.9	C21—C22—C23	119.7 (3)
C5—C6—C7	120.6 (3)	C21—C22—H22	120.1
C5—C6—H6	119.7	C23—C22—H22	120.1
C7—C6—H6	119.7	C24—C23—C18	119.9 (3)
C6—C7—C8	118.4 (3)	C24—C23—C22	119.7 (3)
C6—C7—C9	120.5 (3)	C18—C23—C22	120.4 (3)
C8—C7—C9	121.1 (2)	N4—C24—C23	178.5 (4)
C7—C8—C3	122.0 (3)	N1—C25—C26	114.0 (2)
C7—C8—H8	119.0	N1—C25—H25A	108.8
C3—C8—H8	119.0	C26—C25—H25A	108.8
N1—C9—C7	113.2 (2)	N1—C25—H25B	108.8
N1—C9—H9A	108.9	C26—C25—H25B	108.8
C7—C9—H9A	108.9	H25A—C25—H25B	107.7
N1—C9—H9B	108.9	C27—C26—C31	117.2 (3)
C7—C9—H9B	108.9	C27—C26—C25	122.3 (2)
H9A—C9—H9B	107.7	C31—C26—C25	120.5 (2)
N1^i^—C10—C11	112.9 (2)	C26—C27—C28	121.6 (3)
N1^i^—C10—H10A	109.0	C26—C27—H27	119.2
C11—C10—H10A	109.0	C28—C27—H27	119.2
N1^i^—C10—H10B	109.0	C29—C28—C27	119.9 (3)
C11—C10—H10B	109.0	C29—C28—H28	120.1
H10A—C10—H10B	107.8	C27—C28—H28	120.1
C12—C11—C16	117.5 (2)	C30—C29—C28	119.9 (3)
C12—C11—C10	120.0 (2)	C30—C29—H29	120.0
C16—C11—C10	122.4 (2)	C28—C29—H29	120.0
C13—C12—C11	120.9 (3)	C29—C30—C31	120.0 (3)
C13—C12—H12	119.6	C29—C30—H30	120.0
C11—C12—H12	119.6	C31—C30—H30	120.0
C14—C13—C12	122.0 (3)	C30—C31—C26	121.4 (3)
C14—C13—H13	119.0	C30—C31—C32	118.1 (3)
C12—C13—H13	119.0	C26—C31—C32	120.6 (3)
C13—C14—C15	117.1 (2)	N3—C32—C31	179.3 (3)
C13—C14—C1	122.2 (2)	C25—N1—C10^i^	110.5 (2)
C15—C14—C1	120.7 (3)	C25—N1—C9	109.9 (2)
C14—C15—C16	121.3 (3)	C10^i^—N1—C9	111.2 (2)
C14—C15—H15	119.4	C17—N2—C1	110.6 (2)
C16—C15—H15	119.4	C17—N2—C2	109.7 (2)
C11—C16—C15	121.2 (2)	C1—N2—C2	109.6 (2)
			
N2—C2—C3—C4	116.7 (3)	C18—C19—C20—C21	−1.9 (5)
N2—C2—C3—C8	−66.8 (3)	C19—C20—C21—C22	−0.1 (5)
C8—C3—C4—C5	0.1 (4)	C20—C21—C22—C23	0.5 (5)
C2—C3—C4—C5	176.6 (3)	C19—C18—C23—C24	178.6 (3)
C3—C4—C5—C6	0.0 (5)	C17—C18—C23—C24	−3.6 (4)
C4—C5—C6—C7	0.1 (5)	C19—C18—C23—C22	−2.9 (4)
C5—C6—C7—C8	−0.3 (4)	C17—C18—C23—C22	174.9 (3)
C5—C6—C7—C9	178.2 (3)	C21—C22—C23—C24	179.5 (3)
C6—C7—C8—C3	0.3 (4)	C21—C22—C23—C18	1.1 (5)
C9—C7—C8—C3	−178.2 (2)	N1—C25—C26—C27	−27.7 (4)
C4—C3—C8—C7	−0.2 (4)	N1—C25—C26—C31	154.1 (2)
C2—C3—C8—C7	−176.9 (2)	C31—C26—C27—C28	−1.0 (4)
C6—C7—C9—N1	145.3 (2)	C25—C26—C27—C28	−179.3 (3)
C8—C7—C9—N1	−36.2 (3)	C26—C27—C28—C29	0.1 (5)
N1^i^—C10—C11—C12	−151.7 (2)	C27—C28—C29—C30	0.9 (5)
N1^i^—C10—C11—C16	31.4 (4)	C28—C29—C30—C31	−0.8 (5)
C16—C11—C12—C13	0.8 (4)	C29—C30—C31—C26	−0.3 (4)
C10—C11—C12—C13	−176.3 (3)	C29—C30—C31—C32	179.3 (3)
C11—C12—C13—C14	−0.1 (5)	C27—C26—C31—C30	1.2 (4)
C12—C13—C14—C15	−0.8 (4)	C25—C26—C31—C30	179.4 (2)
C12—C13—C14—C1	177.2 (3)	C27—C26—C31—C32	−178.4 (3)
N2—C1—C14—C13	109.1 (3)	C25—C26—C31—C32	−0.1 (4)
N2—C1—C14—C15	−72.9 (3)	C26—C25—N1—C10^i^	−72.3 (3)
C13—C14—C15—C16	1.0 (4)	C26—C25—N1—C9	164.6 (2)
C1—C14—C15—C16	−177.0 (3)	C7—C9—N1—C25	−74.6 (3)
C12—C11—C16—C15	−0.6 (4)	C7—C9—N1—C10^i^	162.7 (2)
C10—C11—C16—C15	176.4 (3)	C18—C17—N2—C1	165.5 (2)
C14—C15—C16—C11	−0.3 (5)	C18—C17—N2—C2	−73.5 (3)
N2—C17—C18—C19	−39.2 (4)	C14—C1—N2—C17	−65.6 (3)
N2—C17—C18—C23	143.0 (3)	C14—C1—N2—C2	173.4 (2)
C23—C18—C19—C20	3.4 (4)	C3—C2—N2—C17	161.1 (2)
C17—C18—C19—C20	−174.5 (3)	C3—C2—N2—C1	−77.4 (3)

Symmetry codes: (i) −*x*+1, −*y*+1, −*z*+1.

### Hydrogen-bond geometry (Å, °)

**Table:**

*D*—H···*A*	*D*—H	H···*A*	*D*···*A*	*D*—H···*A*
C30—H30···Cg1^ii^	0.93	2.60	3.451 (4)	152
C2—H2A···Cg2^iii^	0.97	2.97	3.937 (4)	177

Symmetry codes: (ii) −*x*, −*y*, −*z*+2; (iii) *x*, *y*+1, *z*.
